# Two cases of infantile-onset primary generalized glucocorticoid hypersensitivity and the effect of mifepristone

**DOI:** 10.1186/s12887-022-03722-3

**Published:** 2022-11-08

**Authors:** Xiu Zhao, Zhongwei Xu, Huiping Su, Rongfei Zheng, Min Zhan, Yuge Huang, Zhe Su

**Affiliations:** 1 Department of Endocrinology, Shenzhen Children’s Hospital, 7019# Yitian Road, Futian District, Shenzhen, 518038 Guangdong Province China; 2 Pharmacy Department, Shenzhen Children’s Hospital, Shenzhen, 518000 Guangdong Province China; 3 Department of Pediatrics, the Affiliated Hospital of Guangdong Medical University, Zhanjiang, 524023 Guangdong China

**Keywords:** Primary generalized glucocorticoid hypersensitivity, Cushing syndrome, Glucocorticoids, Mifepristone

## Abstract

**Background:**

Primary generalized glucocorticoid hypersensitivity (PGGH) is a very rare disease caused by terminal organ hypersensitivity to glucocorticoids for which the aetiology is unknown. The incidence of PGGH is extremely rare, especially in children. To date, the literatures about the etiology, prognosis and treatment of PGGH are scarce. Aim of the study is describing the cases of two Chinese children with infantile-onset PGGH in one family, one of whom died and one who was treated with mifepristone. They are the two youngest children with PGGH reported in the literature.

**Case presentation:**

Two siblings with infantile-onset PGGH were affected in this family. The main manifestations of patient 1 were typical Cushing’s syndrome-like manifestations, significantly aggravated symptoms after physiological doses of glucocorticoids and very low levels of serum cortisol and adrenocorticotropin hormone (ACTH) during attacks. After being diagnosed with PGGH, he was given guidance to avoid glucocorticoids and took mifepristone therapy for 5 months, and his symptoms improved. Patient 2 was the younger brother of patient 1, with similar manifestations to his brother at the age of 4 months. Patient 2 ultimately died at the age of 9 months.

**Conclusion:**

PGGH is a very rare disease that can lead to death if not diagnosed and treated in a timely manner. This article describes the cases of the two youngest children with PGGH reported in the literature, one of whom improved after mifepristone treatment, and increases the knowledge of the clinical manifestations of and the treatment experience in PGGH.

## Background

Primary generalized glucocorticoid hypersensitivity (PGGH) is a disease caused by terminal organ hypersensitivity to glucocorticoids for which the aetiology is unknown [1]. PGGH is characterized by an overreaction of target organs to corticosteroids, which is characterized by the presence of Cushing’s syndrome-like manifestations and normal/low blood cortisol and adrenocorticotropin hormone (ACTH) levels [2]. The incidence of PGGH is extremely rare. To date, only 16 cases have been reported, of which only 4 were children [3–6]. At the same time, there are few reports on the treatment and prognosis of PGGH. This paper reports the cases of two children with infantile-onset PGGH in one Chinese family, one of whom was treated with mifepristone.

## Case presentation

**Fig. 1:**
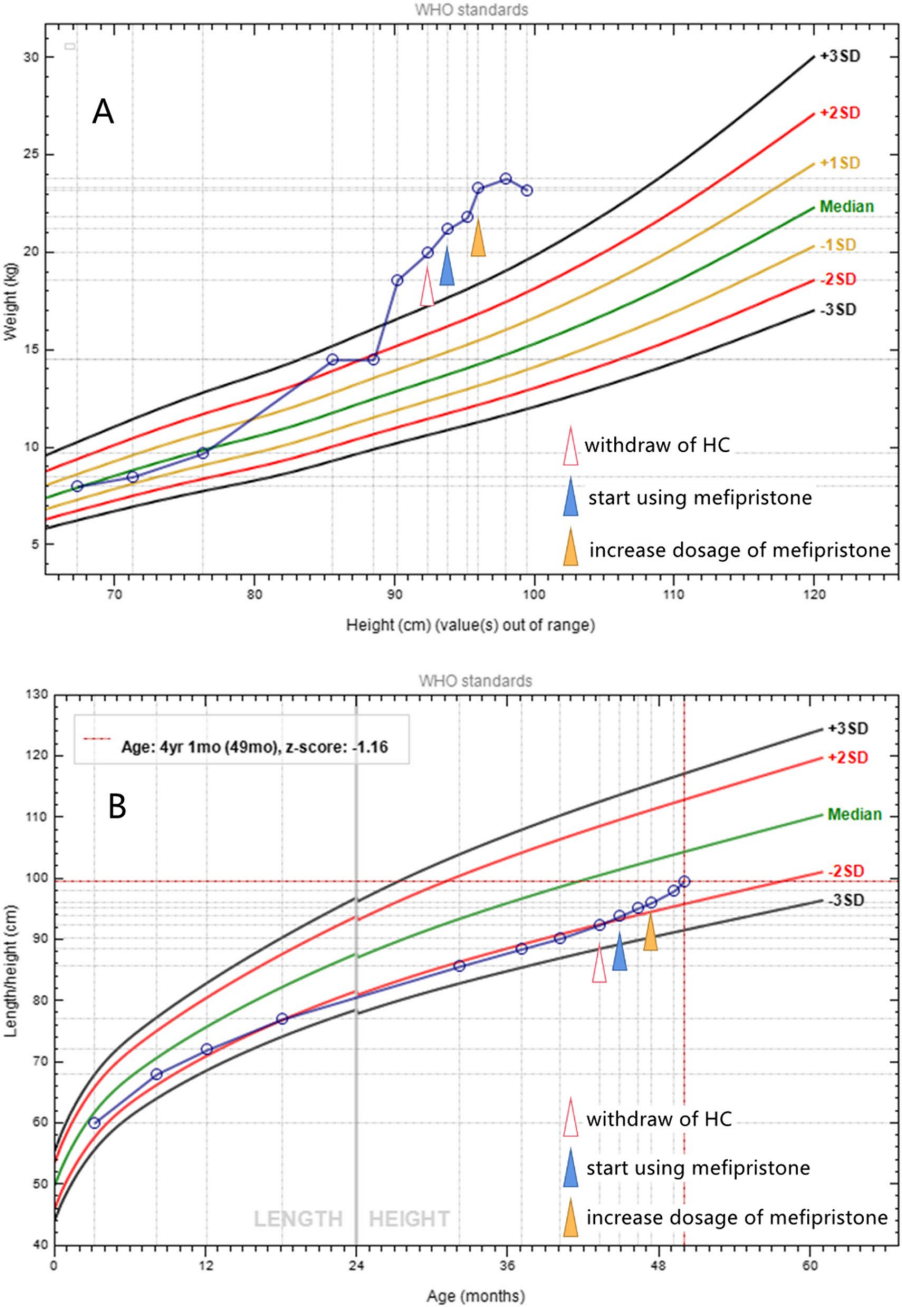
The growth pattern of patient 1 before and during mifepristone treatment. HC: hydrocortisone. **A** Weight-for-height curves (WHO). **B** Height-for-age curves (WHO)

**Fig. 2:**
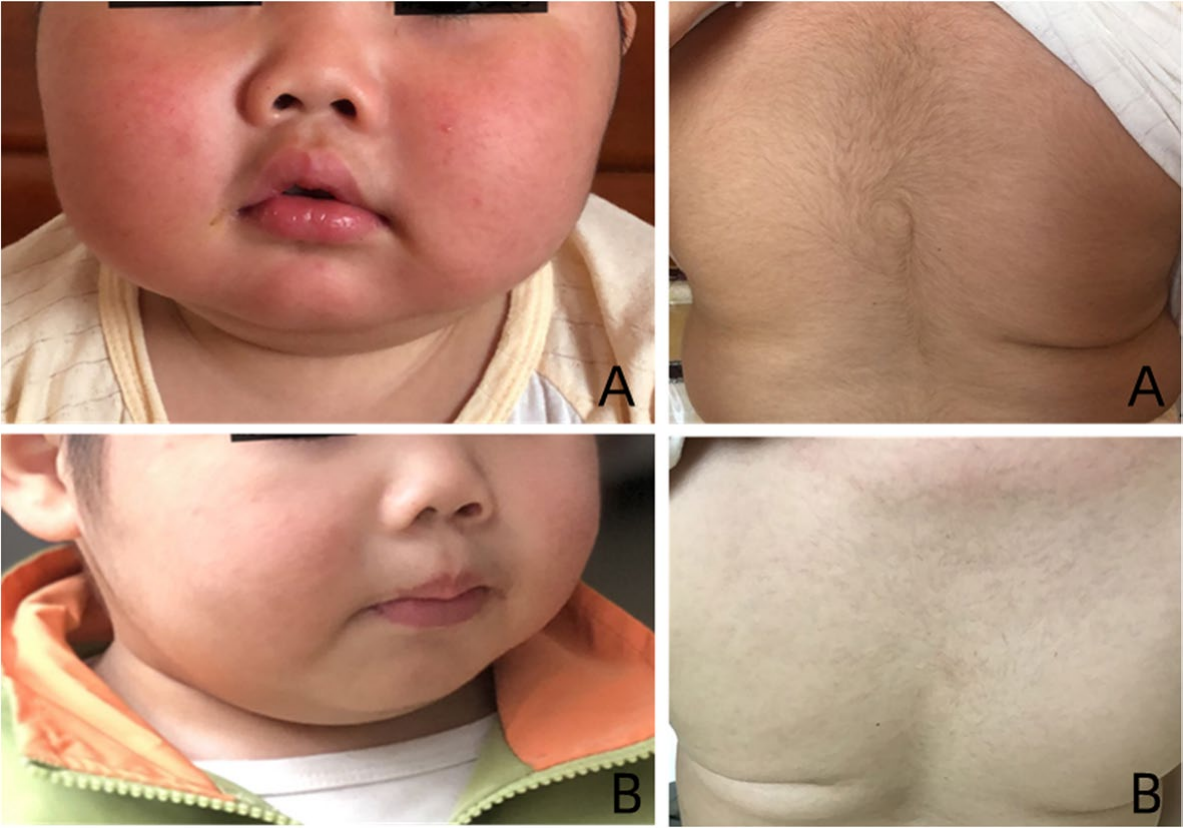
The change in Cushing’s syndrome-like manifestations in patient 1 before and during treatment with mifepristone. **A** Before the treatment; **B** After 5 months of treatment

Patient 1 (see Figs. 1 and 2 and Table 1), the proband, was a 3-year and 7-month-old boy. He was admitted because of growth retardation for 3 years and rapid weight gain for more than 2 years. Since the age of 8 months, he has been stunted, with a low height standard deviation (SD) score from − 1.25 SD to − 2.04 SD. His weight increased rapidly after the age of 2 years. During the age of 2–3 years, his weight gain varied regularly, with 1–2 weeks of rapid gain followed by 2–4 weeks of slower gain. After the age of 3 years, his weight increased continuously, from − 0.24 SD to + 4.89 SD (weight-to-height standard score), and he had hirsutism, a moon face and acne, fatigue, reduced physical capacity and decreased cognitive ability. In an external hospital, he was diagnosed with adrenal insufficiency because of the significantly decreased serum cortisol and ACTH and given hydrocortisone (HC) 7.5 mg (9.38 mg/m^2^/d). During the 2-month HC treatment, the above symptoms were further aggravated.

**Table 1:**
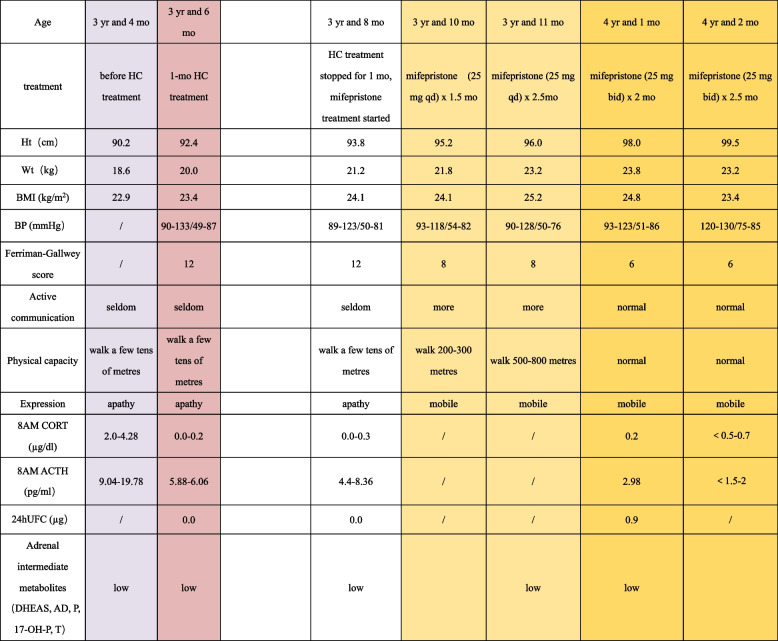
The data of patient 1 before and during mifepristone treatment

*HC* Hydrocortisone, *Ht* Height, *Wt* Weight, *BP* Blood pressure, *BMI* Body mass index, *ACTH* Adrenocorticotropin hormone, 24hUFC: 24-hour urine free cortisol, *yr* year, *mo* month, *AD* Androstenedione, *DHEAS* Dehydroepiandrosterone, *T* Testosterone, *P* Progesterone, *17-OH-P* 17-hydroxyprogesterone. The blue part indicates the data before hydrocortisone treatment. The red part indicates the data during hydrocortisone treatment. The white part indicates the data after stopping hydrocortisone treatment. The yellow part indicates the data during the mifepristone treatment. The light and dark yellow parts indicate different dosages of mifepristone (25 mg qd and 25 mg bid) treatment

**Fig. 3:**
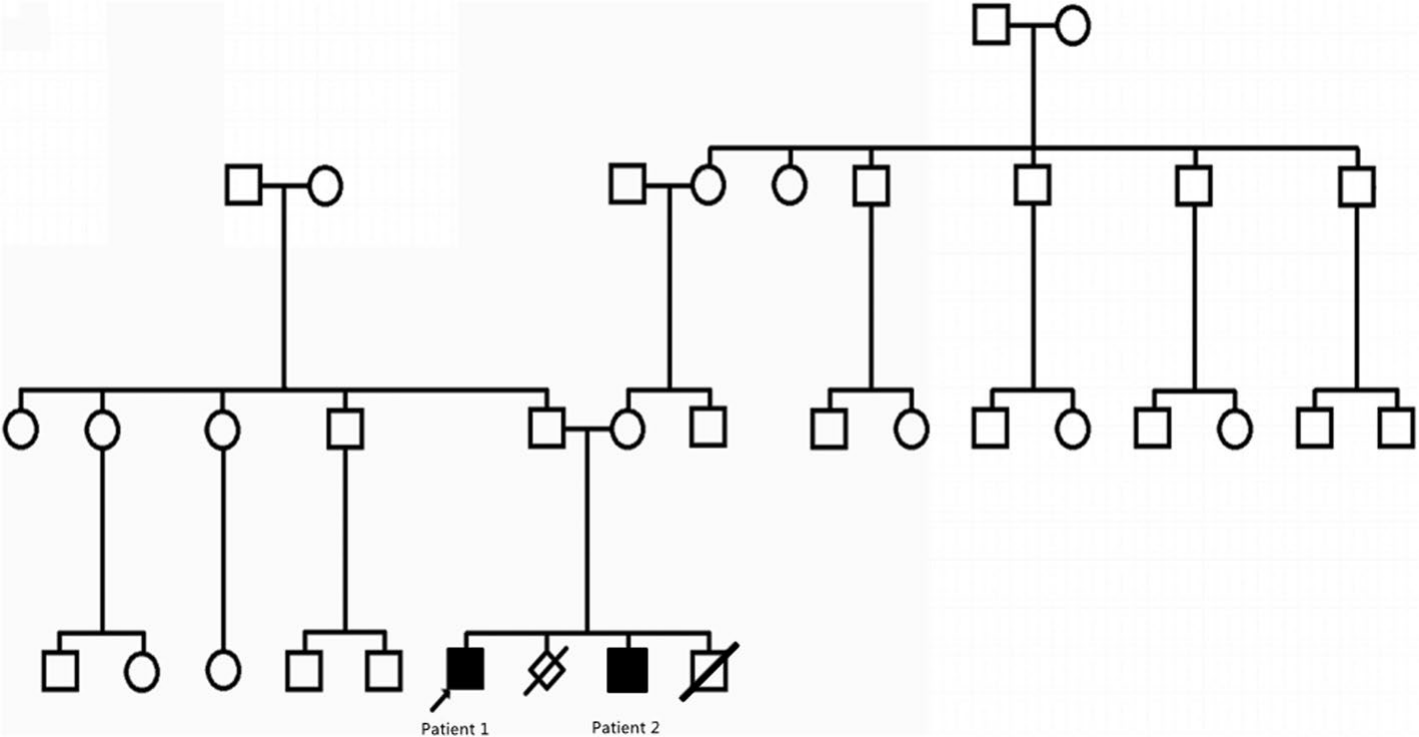
The pedigree of patient 1 and patient 2

As the first child of nonconsanguineous parents, he was born at 38 + 5 weeks of gestation via vaginal delivery, and his birth weight was 3.3 kg. He had suffered from oral *Candida* infections and pneumonia. No abnormalities in the history of birth, feeding, psychomotor development, operations or vaccinations were reported. The patient had two younger brothers. The elder younger brother is patient 2. The other brother died of an intracranial haemorrhage at the age of 1 week without manifestations similar to his siblings. His mother had a spontaneous miscarriage at the first trimester of her second pregnancy (see Fig. 3).

Physical examination: Height, 92.4 cm (− 2.04 SD); weight, 20 kg (weight-to-height standard score + 4.89 SD); body mass index (BMI), 23.43 kg/m^2^ (> 97th percentile (P97th) =17.6); and blood pressure (BP), 90–133/49–87 mmHg (83.3% systolic blood pressure (SBP) > P95th, 56.3% diastolic blood pressure (DBP) > P95th). He was expressionless. He showed symptoms of Cushing’s syndrome, such as central obesity, a full moon and flushed, ruddy face, acne, a buffalo hump, and hirsutism (Ferriman-Gallwey score: 12) without striae or axillary hair. He had bilateral knee valgus with 5 cm of ankle spacing. He had normal male external genitalia, with Tanner stage 1. There were no abnormalities of the heart, lung, abdomen, muscle strength, or muscle tension.

Laboratory examination: The results of complete blood count, routine urine, routine faecal, liver and kidney function, carcinoembryonic antigen (CEA), alpha-fetoprotein (AFP), neuron-specific enolase (NSE), human chorionic gonadotropin (HCG), atrial natriuretic peptide and thyroid function analyses were normal. High-density lipoprotein cholesterol was 0.94 mmol/L. The levels of IGF-1 and IGFBP3 were 291 ng/ml (78.2 ± 31.2 ng/ml) and 3.9 μg/ml (1.99 ± 0.5 μg/ml), respectively. The glucose tolerance test result was normal. Before the usage of HC, 8 AM cortisol and ACTH were 2.0–4.28 μg/dl (reference range 5–23 μg/dl) and 9.04–19.78 pg/ml (10–80 pg/ml), respectively. The levels of androstenedione (AD), dehydroepiandrosterone (DHEAS), testosterone (T), progesterone (P) and 17-hydroxyprogesterone (17-OH-P) were lower than normal. The levels of renin activity, angiotensin II and aldosterone were normal. After the withdrawal of HC, the levels of serum 8 AM cortisol and ACTH and 24-hour urinary free cortisol (UFC) were undetectable. His parents’ serum 8 AM ACTH and cortisol levels were normal. No abnormalities were found on electrocardiography (ECG) or ultrasonography of the heart, liver, gallbladder, pancreas, spleen, urinary system, adrenal gland, retroperitoneum, abdominal aorta, renal artery, or carotid artery. No abnormality was found in the magnetic resonance imaging (MRI) of the brain and pituitary. The whole-genome sequencing found no pathogenic or possibly pathogenic mutations and no copy number variations or chromosome abnormalities related to the clinical manifestations of the patient. No pathogenic gene mutations related to the phenotype were found in the patient’s mitochondrial genome.

Diagnosis, Treatment and follow-up: The patient stopped taking HC immediately and avoided contact with any form of glucocorticoid. He was given diet and exercise management and antihypertension treatments. After the 1-month follow-up, the patient’s weight still increased rapidly, and his hypertension, hirsutism, acne and fatigue did not improve. The 8 AM cortisol and ACTH and 24-hour UFC levels were still lower than normal. He was diagnosed as PGGH according to excessive glucocorticoid manifestations and the low serum cortisol and ACTH. After discussion with pharmaceutical experts, oral treatment with mifepristone 25 mg once a day (1.2 mg/kg/d) was started. After 2.5 months of mifepristone treatment, the patient’s height increased 2.2 cm, and his activity, exercise capacity, acne and hirsutism significantly improved,.but no obvious change in BP was observed. Due to continued weight gain and physical capacity problems, mifepristone was given orally at a dosage of 25 mg twice a day. After 5 months of mifepristone treatment, his weight and BMI began to decrease, accompanied by good growth (5.7 cm/5 months). His acne disappeared, and his hirsutism improved. He was full of energy, similar to a healthy boy. His parents were satisfied with the current treatment effect.

**Fig. 4:**
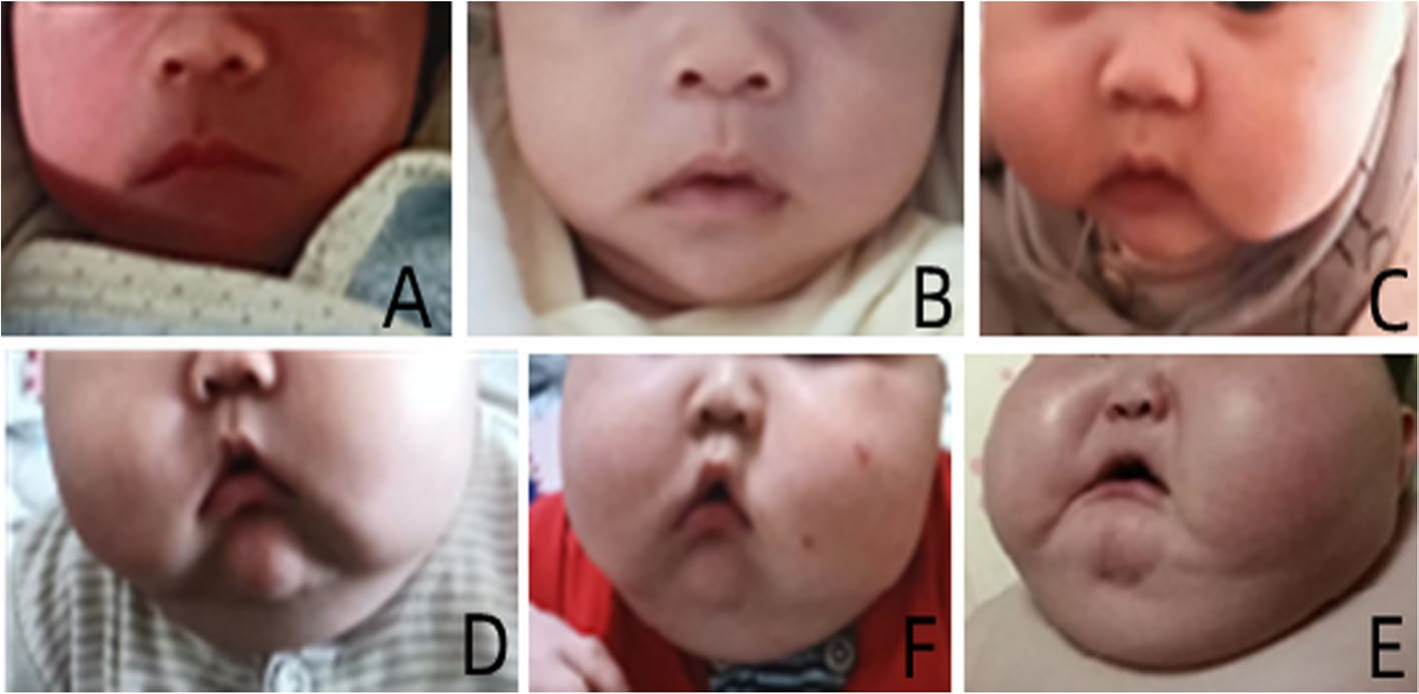
The data of patient 2 from the age of 8 days to 9 months. **A** 8 days old. **B** 2 months old. **C** 4 months old before treatment with mometasone ointment. **D** 5 months old during the treatment with mometasone ointment. **E** 6.6 months old without treatment with mometasone ointment. **F** 8 months old after hydrocortisone treatment

**Fig. 5:**
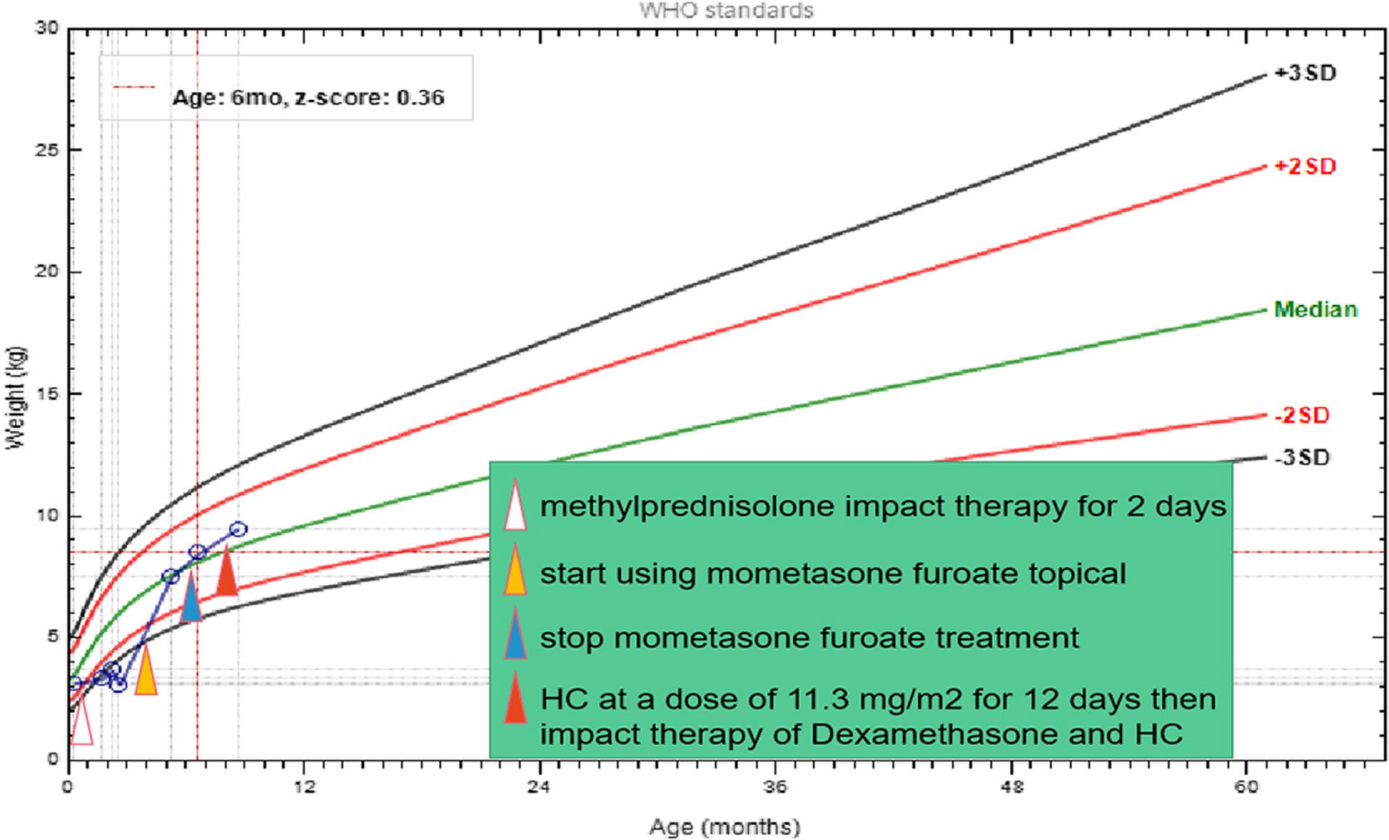
The weight of patient 2 from the age of 8 days to 9 months

Patient 2 (see Figs. 4 and 5) was a younger brother of the proband. He was born at 38 weeks of gestation via vaginal delivery, and his birth weight was 3.3 kg. At the age of 8 days, he was admitted because of necrotizing enterocolitis and sepsis, for which he was treated with methylprednisolone for 2 days. At 1.6 months after birth, exploratory laparotomy and partial ileectomy were performed because of ileal perforation. Intestinal obstruction with malnutrition was relieved by conservative treatment. At the age of 4 months, a small amount of mometasone ointment was used because of rash. After that, he experienced rapid weight gain and slowed growth. His weight increased 5.44 kg over the next 4 months without extra food intake. At the ages of 5.3 months and 6.6 months, respectively, he was admitted because of severe obesity. At the age of 6.6 months, hypertension (BP 100–110 68 mmHg) and low cortisol (0.42 μg/dl) were found. Mometasone ointment was discontinued, and HC (11.3 mg/m^2^/d) was given orally in consideration of secondary adrenocortical insufficiency. At the age of 8 months, he was admitted to the PICU because of anhelation and severe obesity. Physical examination: Temperature, 36.7 °C; pulse, 172 beats/min; respiratory rate, 70 times/min; BP, 143/91 mmHg; weight, 9.5 kg (weight-to-height standard score + 3.23 SD); length, 64 cm (− 3.04 SD); head circumference, 42.5 cm; oxygen saturation (sPO_2_), 88%; obese; a fullmoon and flushed, ruddy face; and a buffalo hump. The breathing sounds of both lungs were coarse, and no rales were heard. The heart rate was 172 beats per minute, but no gallop rhythm or murmur were heard. An old surgical scar could be seen in the middle of the abdomen, but no other abnormalities were found in the physical examination. Laboratory examination: The level of 8 AM cortisol (0.42–1.46 μg/dl), ACTH (< 5.05 pg/ml), DHEAS, AD and T were lower than the normal values. Liver and kidney function, electrolytes, glucose, CEA, AFP, HCG, NSE and ECG findings were normal. The results of ultrasonography of the heart, liver, gallbladder, pancreas, spleen, urinary system, and both adrenal glands were normal. No abnormality was found by abdominal computed tomography (CT). Unfortunately, the boy died at the age of 9 months with the corresponding treatments including dexamethasone and HC. At last he also was diagnosed as PGGH according to Cushing’s syndrome-like manifestations and the low serum cortisol and ACTH.

## Discussion and conclusions

PGGH is a very rare and lethal disease for which there is limited experience in treatment. This article describes the cases of 2 children with infantile-onset PGGH in a Chinese family. The two siblings are the youngest patients with PGGH reported thus far. Patient 2 died, and patient 1 improved with mifepristone treatment.

Here, we presented the cases of two boys with infantile-onset excessive glucocorticoid manifestations [3], including central obesity, Cushing’s syndrome-like symptoms, and growth retardation with significant weight gain. As a history of contact with alcohol or viral infections was excluded, the low serum cortisol and ACTH may have been due to periodic Cushing’s syndrome or PGGH. Because there was no increase in cortisol even during the clinical exacerbation period, periodic Cushing’s syndrome could be excluded. Therefore, on the basis of the aggravation of symptoms after the use of low-dose or even physiological doses of glucocorticoids in the past, the diagnosis of PGGH was confirmed.

The spectrum of sensitivity to glucocorticoids in the population is continuous [7]. During glucocorticoid therapy in patients with congenital adrenocortical hyperplasia, nephrotic syndrome or rheumatoid arthritis, sensitivity to glucocorticoids shows some individual differences [8]. The extremes of the spectrum are glucocorticoid hypersensitivity and glucocorticoid insensitivity syndrome (GIS). There are many factors affecting glucocorticoid sensitivity, including genomic effects and nongenomic effects. These factors include changes in the bioavailability of glucocorticoids, the concentration of corticosteroid binding globulin, the balance of *11βHSD1* and *11βHSD2* activity, multidrug resistance (MDR) pump activity and its gene polymorphisms, an increase in glucocorticoid receptor (GR) α, the enhanced binding ability of GRα to glucocorticoids, the activation of NF-κB in the post-GR receptor pathway, abnormal cytokines/molecular chaperones regulating GR action, and GR gene mutations/polymorphisms [8, 9]. Abnormalities related to GR are the most likely pathogenesis of PGGH. Glucocorticoids act mainly through GR, which is encoded by the *NR3C1* gene. After variable splicing of exon 9, this gene forms GRα and GRβ. GRα is widely expressed and binds to glucocorticoids, while GRβ does not bind to glucocorticoids but has a negative effect on GRα [10]. Laboratory studies have found that GRβ can form a dimer with GRα and directly regulate the expression of downstream genes [11–13].

It is difficult to identify the aetiology of PGGH, which can be found in only a few case studies. To date, studies in patients have found that possible causes include infection (such as rubella infection) [1], abnormal thermal stability and hGR affinity [14], an increase in hGR with normal affinity [3], an abnormal NF-κB response in the GR postreceptor signal transduction pathway [4] and abnormal gene levels. The possible mechanisms that cause PGGH at the genetic level include the increased GR sensitivity due to the *NR3C1* gene polymorphisms p.N363S and Bcl1; the p.D401H mutation in the *NR3C1* gene, which can lead to tissue-selective glucocorticoid hypersensitivity; and the p.G3134T mutation in the *NR3C1* gene, which can lead to systemic glucocorticoid hypersensitivity [15–20]. While no mutation was found in either patient in this report. Further studies on GR should be considered.

There are few reports on the prognosis of PGGH (see Table 2). Three untreated patients with PGGH were in spontaneous remission [1, 4], but there were also deaths, as seen in patient 2. Despite guidance to follow a strict diet and proper exercise to control weight and to avoid any contact with glucocorticoids, the symptoms and laboratory parameters of patient 1 were still aggravated. Given that his PGGH symptoms were not temporary and the death of his younger brother, patient 1 needed to be treated as soon as possible.

**Table 2:** The data of the cases with PGGH available in the literature

Author	case	Age (year)	Gender	Ethnicity	Family history	GC exposure	Clinical manifestation	Cortisol (nmol/L)	ACTH (pmol/L)	24hUFC (nmol/24 h)	Imaging of adrenal gland	Treatment	Prognosis
Our case	1 (Patient 1)	3.6	M	Chinses	+	+	central obesity, hirsutism, moon face, acne, buffalo hump, fatigue, decreased cognitive ability, hypertension	0.00–117.27	0.00–4.35	undetectably low	normal	mifepristone	remission
	2 (Patient 2)	0.55	M	Chinses	+	+	central obesity, moon face, acne, buffalo hump, hypertension	11.51–40	<1.00		normal	no	death
Russcher et al. [3]	3	13	F	Netherlander	–	+	obesity, fatigue, growth retardation, violaceous striae, osteopenia	<30		<3.00	normal	stop using budesonide	spontaneous remission
Newfield et al. [5].	4	10.8	F	Americian	–	–	central obesity, moon face, buffalo hump, violaceous striae, osteopenia, learning disability, early puberty	304.18 ± 15.28	6.09 ± 2.35	normal	normal	mifepristone	remission
Nicolaides et al. [4].	5	9	F	Greek	–	–	central obesity, moon face, buffalo hum, violaceous striae, acanthosis nigricans, hirsutism	0.45–7.79	1.00	9.00	normal	no	spontaneous remission
Su, et al. [6]	6	child	NM	Chinses	–	–	central obesity, moon face, buffalo hum, violaceous striae, hirusm, hypertension, growth retardation, ostalgia	Low	Normal	Low	normal	NM	NM
Iida et al. [14]	7	54	M	Japanese	+	–	central obesity, moon face, buffalo hump, DM	20.00 ± 19.00	<2.00	40.00–50.00	normal	NM	NM
Krysiak et al. [21]	8	28	F	Polish	–	–	Obesity, hypertension, prediabetes, osteopenia	120.31	<2.00	272.4–317.8	adrenal gland atrophy	ketoconazole, cabergoline	death because of an accident
Liu., et al. [22]	9	27	M	Chinses	+	–	central obesity, moon face, buffalo hum, violaceous striae, osteopenia	10.75	<0.22	11.95	adrenal gland atrophy	mifepristone	remission
	10 (father of case 9)	adult	M	Chinses	+	–	hypertension, moon face, buffalo hum, violaceous striae, osteopenia, hyperglycemia	NM	< 0.22	23.62	normal	no	exacerbation
Al-Shoumer, et al. [23]	11	32	F	Kuwaitis	–	+	obesity, moon face, buffalo hum, violaceous striae, DM, renal calculus, myasthenia, hypertension,	28	< 1.00	undetectably low concentration	adrenal myelolipoma	NM	NM
Zhang, et al. [1]	12	29	M	Chinses	–	–	central obesity, moon face, buffalo hum, violaceous striae, acne, osteopenia, hypokalemia, impaired glucose tolerance, hypertension	72.64–216.56	5.60–9.56	378.12	normal	no	spontaneous remission
Santen, et al. [17]	13	46	F	Americian	+	+	obesity, moon face, fatigue, headache, abdominal pain, nausea, diarrhea, anxiety/depression, muscle and joint aches, hypertension	63.00–98.00	NM	276.00	NM	no	NM

*GC* Glucocorticoid, *F* Female, *M* Male, *DM* Diabetes mellitus, *NM* No mention

To date, only 3 patients with PGGH were treated with drugs (see Table 2). Because of the low level of cortisol in blood, mifepristone was the first choice to act on GR. Mifepristone has been approved by the Food and Drug Administration (FDA) and several international guidelines for the treatment of Cushing’s syndrome [9, 24, 25]. Mifepristone has a similar structure to progesterone and glucocorticoids and works by selectively antagonizing progesterone receptors at low doses and antagonizing GR at high doses. Mifepristone can act on GRα and GRβ and directly regulate GRβ gene expression independent of GRα [26]. The affinity of mifepristone is 18 times that of cortisol, and mifepristone can effectively improve the clinical syndrome caused by hypercortisolism. In addition to an adult female patient with PGGH who used ketoconazole because mifepristone was not available [21], a 27-year-old male patient and a 13.75-year-old girl with PGGH were treated with mifepristone, and their symptoms were improved [5, 22]. The experience with mifepristone in children is very limited. Patient 1 is the youngest patient with PGGH reported to be treated with mifepristone. Due to the experience of mifepristone treatment in the adult patients with PGGH and Cushing’s syndrome, the dosage of mifepristone in PGGH should be lower than that administered for Cushing’s syndrome. Therefore, the initial dose administered for patient 1 was 1.2 mg/kg/d (25 mg qd). The dose was gradually increased according to the situation during the follow-up, and the adverse reactions of mifepristone were monitored [20]. Within 5 months of treatment, his clinical manifestations were relieved. His height developed well, and his weight decreased. His physical capacity and activity returned to normal. The only adverse reaction during treatment was mild hypokalaemia.

Our report provides the more information of clincalmanifestations, treatment and prognosis for paediatric patients with PGGH. While the aetiology of PGGH in these two cases still could not be found. The more study of aetiology will be our future research continually.

PGGH is a very rare disease. When a child with obvious cushingoid features in presence of reduced ACTH and cortisol levels without exogenous hormone exposure, PGGH should be diagnosis. It is dangerous to treat the patient with PGGH with hydrocortison or other glucocorticoids. PGGH can be lethal without timely diagnosis and correct treatment. This article described the cases of the two youngest children with PGGH reported in the literature, one of whom was the youngest patient treated with mifepristone, and increases the knowledge of the clinical manifestations of and the treatment experience in PGGH.

## Data Availability

The dataset analyzed in the current study is available from the corresponding author upon reasonable request.

## Abbreviations

ACTH: Adrenocorticotropin hormone
AD: Androstenedione
AFP: Alpha-fetoprotein
BP: Blood pressure
CEA: Carcinoembryonic antigen
DBP: Diastolic blood pressure
DHEAS: Dehydroepiandrosterone
ECG: Electrocardiography
FDA: Food and Drug Administration
GIS: Glucocorticoid insensitivity syndrome
GR: Glucocorticoid receptor
HC: Hydrocortisone
HCG: Human chorionic gonadotropin
MDR: Multidrug resistance; MRIMagnetic resonance imaging
NSE: Neuron-specific enolase
P: Progesterone
PGGH: Primary generalized glucocorticoid hypersensitivity
SD: Standard deviation
SBP: Systolic blood pressure
sPO2: Oxygen saturation
T: Testosterone
UFC: Urinary free cortisol
17-OH-P: 17-hydroxyprogesterone
